# Medical Department of Iowa University

**Published:** 1853-08

**Authors:** 


### MEDICAL DEPARTMENT OF THE IOWA UNIVERSITY.

The Faculty of the College deem it wholly unnecessary to repeat what has been so often reiterated, touching the superior advantages of this Institution for successful instruction. Well assured that the public regards the School with an interest which finds its basis in the prospects of its future usefulness, and profoundly grateful for the kind wishes of its friends and the aid of a generous public, the Faculty would beg leave to assure all interested, that the question of success is permanently settled, and the enterprise never had brighter prospects before it. They would also say, that no effort will be wanting on their own part to render it all that the fondest hopes of our fellow citizens of the State can desire.

To answer their expectations the more fully, the Faculty have furnished the College with large additions to their appliances for teaching, by supplying each Chair with all the necessary elements of success; so that the Profession is assured that the highest attainments may be secured within our halls. This large addition to our list of material, has been made, this season, by a personal visit of the Dean, to the Eastern cities, so that the sutdents who may compose our classes in the future, will have all the means necessary to complete intstruction.

*University Hospital.*—This humane enterprise, on the part of the city, was conected with the College, in order to derive the benefits of the professional care of the Faculty, and to furnish to the classes an opportunity for clinical instruction. It will in future be so arranged that all the students shall enjoy, equally and frequently, the advantages to be derived from it. The facilities for practical Anatomy will be, as heretfore, abundantly ample. By a new arrangement, the benefits of this department will be superior to those heretofore enjoyed, and we hope to give it the prominence its importance demands.

*Scholarships.*—It is a fact, better known to physicians in the State than those at a distance, that there are, throughout the State, numerous undergraduate physicians, who have heretofore been deterred, by various causes, from completing the prescribed course of medical study, and who in some instances are at present unable to attend the full courses of lectures, and to defray the somewhat heavy expenses. The thorough education and complete preparation of all such physicians within the State, has been regarded as the most direct and radical means of elevating the character and usefulness of the medical profession. To this end, it has long been a desire of the Faculty to bring about such influences, as would at once induce and enable such practitioners to complete their medical education, and render them thorough and fully competent physicians. Snch a system we are happy to announce has recently been instituted.

It is well known that the State made a generous donation to the College a few years since, for the direct purpose of enlarging the sphere of its professional usefulness. It would seem but a just and proper duty, therefore, on the part of the College, to make some educational return to the State, for the interest it has manifested and the endowment it has made. In accordance, therefore, with the sense of obligation thus engendered, an arrangement has been recently organized, consisting of the establishment of *Scholarships*, certificates of which are lodged with the principal State officers; viz: Governor, Judges, and members of the Legislature. It will be seen that the number of scholarships is neeessarily limited; any medical student, however, desirous of availing himself of the valuable opportunity alluded to, can apply to his local State officers, or address the Faculty.

The scholarships alluded to were issued only a short time since, and already they have met with a friendly reception and decided approval, on the part of all the officers heard from, and an earnest appreciation of the desire of the Faculty. There is also exhibited an enlightened determination to co-operate with it in the extension of its usefulness, and the improvement of the character and attainments of the Medical Profession, throughout the State.

*Preliminary Instruction.*–Desirous of affording every facility to those intending to enter into the profession, and those too who are already pursueing their studies, the Faculty of the College would respectfully invite as many as can make it convenient, to a preparatory course of private instruction, from the 1st of September to the 20th October, when the regular term commences. This will render the usual course, to follow in regular succession, more interesting and profitable, for each branch will receive attention. The offices of the resident Professors will be open for their benefit, and their libraries will be at their service. This prefatory measure is a gratuitous offering on the part of the Faculty, and we doubt not but that the profession will acknowledge its propriety and its advantages. In this way much that must necessarily be omitted in the regular course, can be more fully discussed. Beside this, the student will have acquired a habit of study ; it will aid him in fixing his attention, and he will be better able mentally to digest the mass of instructive matter, comprised in six daily lectures of the regular course.

